# Effects of Creasote on the Economy in Health

**Published:** 1845-12

**Authors:** 


### ARTICLE IV.

### *Effects of Creasote on the Economy in Health.*

Reichenbach has properly remarked that the excessive burning pain in the tongue, which creasote causes, must have at once suggested it to be a poisonous substance. It was soon found that plants, sprinkled with creasote water, died; that fish placed in it were convulsed; and that small animals, as wasps and flies, died when touched with pure creasote. If a small quantity of it be spread upon the hand, and washed off a minute afterwards, the place is found to present a white appearance, but without pain or inflammation. In the course of a few days the place becomes dry, and the cuticle desquamates. When creasote is applied to a part where the epidermis is deficient, or to a wound, instantaneously an extreme violent burning pain is experienced, which continues for eight or ten minutes, but if the part be carefully washed, it gradually ceases. The cause of this is conceived to be the property which creasote possesses of coagulating albumen; and, where blood is flowing, of arresting it. If the rapid disturbance, which it excites, affects important organs, death results sooner or later according to their importance in the economy; relief, however, may be afforded by those substances that dissolve coagulated albumen, as caustic alkalies, acetic acid, &c. It is probable, however, that the poisonous properties result from its acrid character.

To appreciate the physiological effects of creasote, experiments have been undertaken by many individuals. Miguet gave a young dog, for eight days, an ounce a day of distilled water containing four drops of creasote, without any effect. When, however, he doubled the dose, nausea, languor, subsultus tendinum and tremors occurred, followed in the course of a few days, by marked emaciation. On discontinuing the creasote, the functions gradually resumed their pristine condition, and the animal recovered its flesh. To another dog, he gave at once two drams in half an ounce of water, and immediately thereafter great prostration of the muscular system ensued—vertigo, fixed eyes, stupor, dyspnœa, accumulation of mucus in the air passages, spasmodic cough, discharge of large quantities of foamy saliva, with vomiting of a milky matter, although the animal had taken nothing of the kind. After two hours of suffering, the animal died of convulsions. The body was immediately opened; all the tissues, except the liver, exhaled a strong smell of creasote; and the whole of the mucous membrane of the intestinal canal was inflamed. The matters contained in the stomach coagulated when placed in contact with albumen. When heated, they yielded a thick smoke, and a marked smell of creasote. In the heart and large vessels the blood was more firmly coagulated than usual: the lungs were gorged with blood; in the brain there was no evidence either of congestion or hæmorrhage.

In another dog, into whose carotid equal portions of water and creasote were injected, death resulted with similar phenomena, but more rapidly. The precise quantity of creasote used in this experiment is not stated.

Simon, in his experiments, found that when ten drops of creasote, diluted, were injected into a vein, scarcely any effect resulted.

Reiter and Müller, who likewise made experiments on animals, agree with Simon as to the result of injections of creasote into the veins; no special symptoms were induced by it, but this appeared to be owing to the blood being instantaneously coagulated by it, which not only prevented the farther progress of the creasote, but also of the blood, hence no evil consequences resulted; and it is probable, as Riecke has suggested,* that the weaker the solution of creasote, within certain limits, the greater may be its effect on the mass of blood.

Corneliani,† an Italian physician, has also instituted a series of experiments with creasote on lambs, rabbits, &c. All these animals bore small doses of creasote—however unwillingly it might be taken—without any remarkable results, and without loss of appetite. Large doses, however, immediately occasioned general torpor, sudden inclination to pass the urine, paralysis—especially of the lower extremities—with or without convulsions, and frequently the ejection of a bloody foam. When the doses were large, and it was but little diluted, death took place in a few minutes, and on examination, the inner lining of the stomach was generally found corroded, yet not so constantly as to allow of death being ascribed to that circumstance.

It followed, farther, from his experiments, that pure creasote applied to a denuded nerve, or injected only in small quantities into a vein, may occasion death suddenly, and that the application of the creasote to extensive wounded surfaces in the same animals may be ultimately followed by fatal consequences.

Where a very large dose of creasote was administered, immediate death was produced without organic lesion.

In the trials made with it by Dr. Elliotson,‡ he found no action produced upon the bowels; but it sometimes augmented

* Die neuern Arzneimittei, u. s. w. S. 153.

† Giornale delle Scienze Medico-Chirurgiche, No. 8. Febrajo, 1835; Brit. and Foreign Med. Review, p. 265, Jan. 1836, and Journ. de Chimie Medicale, Fev. 1836.

‡ Medico-Chirurg. Transact. vol. xix. London, 1835.

the quantity of urine. He once saw it in doses of a minim three times a day, cause micturition nine times in an hour. In another case, in doses of three minims, it produced severe strangury.

According to Simon, when applied to the muscles, it destroys the surface like a caustic. Müller and Reiter, in their experiments, found that it speedily rendered the muscular fibres of a dirty whitish appearance, and readily lacerable. When applied to the fresh blood of the hog, it converted the color in an instant to an ashy gray; after which it became black and quickly coagulated. Mixed, either pure or diluted, with blood, it thickens it, the mixture assumes a brown red color, and it is found studded with small white points, which are nothing more than coagulated albumen. On exposing the coagulum to the air, it assumes a yellowish red color. Reich, on the other hand, who appears to have made many experiments with creasote, both in internal and external diseases, affirms, that he has never observed any caustic effect from it; from which assertion, as Riecke has remarked,* the only inference to be deduced is, that he must always have applied it largely diluted. Frémanger likewise asserts, that when pure creasote is applied to the epidermis, it does not destroy it; but merely occasions more or less redness of the skin. When applied to a suppurating surface, it caused, instantaneously, the formation of a white pellicle, owing to its coagulating the albumen contained in the secretion from the wound. Adventitious tissues, with which it is brought in contact, are destroyed by it. When placed between the lips of a wound it prevents healing by the first intention, by coagulating the albumen, and, consequently, it may be employed in all cases where it is desirable to prevent the growing together of parts. Frémanger is, indeed, disposed to refer all its efficacy to the action which it exerts on albumen.

Its long continued use often occasions an inflammatory condition which, as Dr. J. L. Da Luz† observes, has nothing in com-

* Op. cit. S. 154.

† Jornal da Sociedade das Sciencas Medicas de Lisboa, tom. v. Lisboa, 1837; reviewed in Zeitschrift fur die gesammte Medicin. Oct. 1838, S. 244.

mon with the disease, for the cure of which it may have been prescribed. In a case of porrigo favosa treated by it recently by the author, febrile irritation supervened, and the head was covered by an artificial eruption, which induced, however, a new action in the intermediate system of the scalp, and after its subsidence, the porrigo was cured.

Dr. Cormack, of Edinburgh, has likewise instituted various experiments on the lower animals to test the physiological effects of creasote.* In three experiments, about twenty-five drops of pure creasote were injected into the venous system of dogs. All the animals died. In every case of poisoning by it, which he has observed, Dr. Cormack found the following to be the symptoms :—Its first deleterious action was a powerful one of sedation on the heart; the vital energies of that organ seeming to be instantaneously paralysed. In some instances, hurried and sonorous respiration went on for more than a minute after the heart had ceased to beat. In general, one or two convulsions, resembling the tetanic, preceded death; and, almost invariably before expiring, the animal uttered one or more shrill cries. In every instance, the atony of the heart immediately after death was very striking.

From other experiments it appears, that when creasote is injected into the arteries, the deleterious effects are of a much milder character, and if the dose is not large the animal may experience but little inconvenience; a circumstance, which proves the importance of a thorough admixture with the blood before the poisonous article reaches the heart; such admixture not taking place, to the necessary extent, when the poison is injected into the veins, but being readily effected when injected into the arteries, and consequently distributed through the capillary or intermediate system.

When taken for any length of time, the urine acquires a blackish hue, and in some cases creasote can be recognised in the urine.†—*Dunglison's New Remedies.*

* Op. cit. p. 66.

† Dr. Macleod, in Medical Gazette, xvi. 559, and xvii. 653.

